# The Comparative Effectiveness of Potent P2Y12 Inhibitors Versus Clopidogrel in Patients with Acute Myocardial Infarction Undergoing PCI: National Registry Data

**DOI:** 10.3390/jcm13216536

**Published:** 2024-10-30

**Authors:** Réka Aliz Lukács, Dániel Tornyos, Péter Kupó, András Jánosi, András Komócsi

**Affiliations:** 1 Heart Institute, Medical School, University of Pécs, 7622 Pécs, Hungary; tornyos.daniel@pte.hu (D.T.); kupo.peter@pte.hu (P.K.); komocsi.andras@pte.hu (A.K.); 2 Hungarian Myocardial Infarction Registry, Gottsegen National Cardiovascular Center, 1096 Budapest, Hungary; andras.janosi@gokvi.hu

**Keywords:** coronary intervention, antiplatelet therapy, mortality, clopidogrel, prasugrel, prediction models, COVID-19, hungarian myocardial infarction registry, high-bleeding risk, elderly patients, decision curve analysis, acute coronary syndromes

## Abstract

Dual antiplatelet therapy (DAPT), which is essential in AMI management, combines aspirin with a P2Y12 receptor antagonist. This study compared the effectiveness of potent P2Y12 inhibitors versus clopidogrel in AMI patients treated with percutaneous coronary intervention (PCI). **Methods:** 65,986 AMI patients included in a nationwide prospective registry who underwent PCI and received DAPT were studied. In total, 9,014 patients received potent P2Y12 inhibitors, and 56,074 received clopidogrel. This study focused on mortality, recurrent myocardial infarction, stroke, repeat revascularization, and major adverse cardiovascular events (MACE) over seven years. The analysis utilized unadjusted models and inverse probability of treatment weighting (IPTW) to compare prognosis, and decision curve analyses were constructed to aid clinical decision making. **Results:** Potent P2Y12 inhibitors significantly reduced mortality risk (unadjusted hazard ratio (HR): 0.58; IPTW HR: 0.68) and MACE (unadjusted HR: 0.66; IPTW HR: 0.78). Diabetic patients showed greater benefits (HR:0.45). In patients at high bleeding risk, the mortality rate was 13% (HR: 0.87, *p* = 0.08). For patients aged 75–79, the HR for mortality was 0.82, whereas for those aged >80 years, it was 0.79, indicating significant mortality risk reduction. Similar trends were observed for MACE. **Conclusion:** This study demonstrated that potent P2Y12 inhibitors are more effective than clopidogrel in reducing mortality and MACE in patients with AMI and underscored their potential role in improving outcomes across diverse patient subgroups. The trend was consistent even during the COVID-19 pandemic. These findings highlight the need for personalized DAPT strategies, particularly for high-bleeding-risk patients, and challenge current guidelines favoring clopidogrel use in older patients.

## 1. Introduction

Despite advancements in therapeutic strategies, acute myocardial infarction (AMI), which encompasses both ST-segment elevation myocardial infarction (STEMI) and non-ST-segment elevation myocardial infarction (NSTEMI), remains a significant contributor to global morbidity and mortality [1]. Central to the management of these conditions is percutaneous coronary intervention (PCI) and dual antiplatelet therapy (DAPT), typically involving aspirin and an adenosine monophosphate (ADP) P2Y12 receptor antagonist. Although improved treatment strategies for myocardial infarction guided by advanced imaging techniques are enhancing the effectiveness of percutaneous coronary intervention, the inhibition of purinergic receptors on platelets represents a significant advancement in cardiology [2]. Dual antiplatelet therapy is essential for the long-term successful application of stent implantation during interventional cardiology procedures. The importance of this mechanism is particularly pronounced in the context of interventions in the prothrombotic environment associated with acute coronary syndrome. However, the selection of P2Y12 inhibitors has been the subject of extensive research and debate [3]. The TRITON-TIMI 38 study, which evaluated prasugrel, a potent P2Y12 inhibitor, in a predominantly PCI-treated patient population, failed to demonstrate a significant mortality benefit when compared to clopidogrel [4]. Conversely, the PLATO trial found that ticagrelor significantly reduced mortality in an acute coronary syndrome (ACS) cohort, including those managed medically without PCI [5]. Adding to the complexity of choice in clinical practice, the ISAR-REACT 5 trial revealed the superiority of prasugrel over ticagrelor in a head-to-head comparison among patients treated with PCI [6].

Current guidelines reflecting these findings advocate for the use of more potent P2Y12 inhibitors—prasugrel or ticagrelor—especially in patients with high thrombotic risk, whereas clopidogrel is reserved for patients in whom these agents are contraindicated or not available. In addition, current recommendations are permissible for the administration of clopidogrel to patients with high age or bleeding risk (HBR). However, real-world adherence to these recommendations is influenced by multiple factors, including cost, availability, and concerns about increased bleeding risk associated with the use of more potent agents [1,7,8].

Clopidogrel remains a key component in DAPT regimens globally, despite a significant decline in its use in the United States in the last decade, where its use has decreased from 87.4% to 28.4%. In contrast, clopidogrel-based DAPT maintains a notable presence. Data from the SCAAR registry revealed that clopidogrel is used in 41% of STEMI cases. This indicates that although its usage patterns may vary, clopidogrel continues to be an integral part of DAPT in many regions globally [9,10].

Consequently, in Hungary, as in many other countries, the adoption of potent P2Y12 inhibitors has been gradual, partially because of these considerations. Considering these dynamics, this study examined the clinical outcomes associated with the use of potent P2Y12 inhibitors versus clopidogrel in a contemporary cohort of patients with AMI.

## 2. Methods

### 2.1. Hungarian Myocardial Infarction Registry (HUMIR)

The registry serves as a comprehensive, prospective, and mandatory project to gather the clinical information of patients hospitalized with AMI within Hungary. Since 2021, it has also been a part of the European Society of Cardiology (ESC) EuroHeart program [11]. Established under the egis of Hungary’s statute CCXLVI. /2013, this internet-based national registry systematically compiles patient data. Previous publications have thoroughly documented the registry’s data acquisition and subsequent follow-up methodologies [12,13].

In essence, HUMIR is an expansive repository encompassing 178 distinct categories that record a wide spectrum of data points. This ranges from baseline demographic information to details regarding coronary intervention. This dataset reflects the healthcare landscape and clinical practices in Hungary [14,15]. The data were processed based on the approval of the central ethics committee. This oversight enabled the current analysis by allowing the use of anonymized data, protecting patient privacy, and enabling a thorough evaluation of treatment outcomes.

### 2.2. Patient Selection and Clinical Endpoints

Our database selection criteria focused on AMI cases in which patients underwent coronary intervention during the acute phase of the event and were initiated on DAPT comprising aspirin (ASA) and a P2Y12 receptor antagonist. P2Y12 inhibitors were selected at the discretion of the treating physician. Patients treated with clopidogrel, prasugrel, or ticagrelor were included in this study. Patients who received potent inhibitors, i.e., prasugrel or ticagrelor, were compared to those who received clopidogrel.

The clinical endpoints evaluated in this study included mortality, subsequent myocardial infarction, stroke, and the necessity for repeat revascularization, either through PCI or coronary artery bypass grafting (CABG). These clinical events (death, new ischemic events, PCI, and CABG) were tracked and recorded using the National Health Insurance Fund database (NEAK). For the current analysis, outcomes within the first 12 months after the intervention were considered.

## 3. Statistical Analysis

Baseline characteristics were summarized using descriptive statistics. Continuous variables are expressed as means with standard deviations (SD) or medians with interquartile ranges (IQR), depending on the normality of the data distribution, whereas categorical variables are presented as frequencies and percentages. Mortality and major adverse events were compared between patients treated with clopidogrel or potent P2Y12-based DAPT using Kaplan–Meier and Cox proportional risk model analyses.

Kaplan–Meier survival curves were generated to illustrate the time-to-event analysis stratified by DAPT type. The log-rank test was used to assess the difference in survival curves. To account for potential confounders and balance potential biases in observational data, propensity score (PS) analysis was performed. A logistic regression model was used to calculate the PS, i.e., the probability of receiving treatment (potent P2Y12 inhibitors), given the covariates listed in Table 1. The covariates included demographic information, vital signs, clinical characteristics, past medical history, and procedures.

After calculating the PS, the inverse probability of treatment weighting (IPTW) was applied using these scores as balancing weights, ensuring that the distribution of observed baseline covariates was independent of treatment assignment. Weighted Cox proportional hazards models were used to evaluate the association between treatment and the endpoints. The absolute risk reduction at 1 year (ARR) was used to calculate the number required to prevent an extra adverse event (NNT).

Our study conducted a comprehensive subgroup analysis to assess the comparative effectiveness of potent P2Y12 inhibitors and clopidogrel. Patients were categorized into specific subgroups based on a range of clinical and demographic factors. These categories encompassed age, sex, diabetes, history of stroke, renal function (classified as severe, moderate, or absent chronic kidney disease), type of ACS (being either STEMI or NSTEMI), and HBR status. For HBR status, both major criteria (such as oral anticoagulant (OAC) use, severe chronic kidney disease, severe anemia) and minor criteria (age over 75 years, moderate chronic kidney disease, moderate anemia, history of stroke) were identified from the database in line with the Academic Research Consortium (ARC) HBR criteria. Patients meeting at least one major or two minor criteria were classified under high bleeding risk. It is important to note that not all 20 criteria outlined in the ARC HBR definition were identifiable in our database [1,2]. Consequently, subgroup analyses were not conducted for patients not categorized as having an HBR. For each subgroup, Cox proportional hazards models were used to compute the hazard ratios (HRs) for 1-year mortality. Additionally, interaction terms were incorporated into the models to explore potential variations in treatment effects among the different subgroups.

A decision curve analysis (DCA) was conducted to evaluate the clinical utility of potent antiplatelet therapy in reducing mortality and MACE. Cox proportional hazards models were fitted, and the predicted risks were calculated for each patient using these models. We also included age as a continuous variable and examined interaction terms between treatment and age to assess potential modifications in treatment effects. Predicted risks from these models were used to create data frames with binary outcomes and predicted risks. DCA was performed at threshold probabilities ranging from 0 to 1 in increments of 0.01, and net benefits were plotted to visualize the effectiveness of treatment decisions based on these models. Separate decision curves were plotted for models including age and interaction terms to determine the impact of age on treatment efficacy.

All statistical analyses were conducted using R 4.2.2, a software environment for statistical computing.

## 4. Results

**Figure 1:**
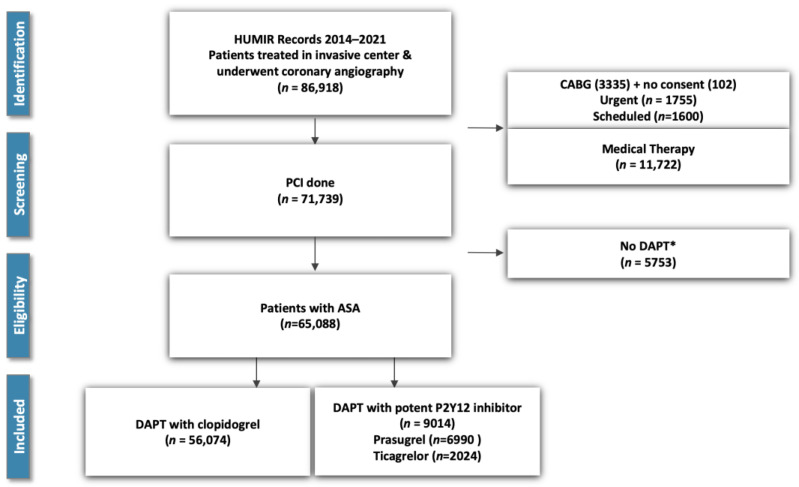
Patient Selection Flowchart. The figure illustrates the record selection from the Hungarian Myocardial Infarction Registry (HUMIR) for the Study of Dual Antiplatelet Therapy in Myocardial Infarction. * DAPT: dual antiplatelet therapy was defined as treatment with low-dose aspirin (ASA) and either clopidogrel or a potent P2Y12 inhibitor (prasugrel or ticagrelor). PCI: Percutan Coronary Intervention, CABG: Coronary Artery Bypass Graft Surgery.

Upon filtering the database records, we identified 65,986 patients who underwent PCI and were administered DAPT in response to an ACS event (the record identification process is depicted in Figure 1).

Of these patients, 9014 received potent P2Y12 inhibitor-based dual antiplatelet therapy and 56,074 received clopidogrel-based DAPT. There were notable differences in patient characteristics between the two groups (Table 1).

**Figure 2:**
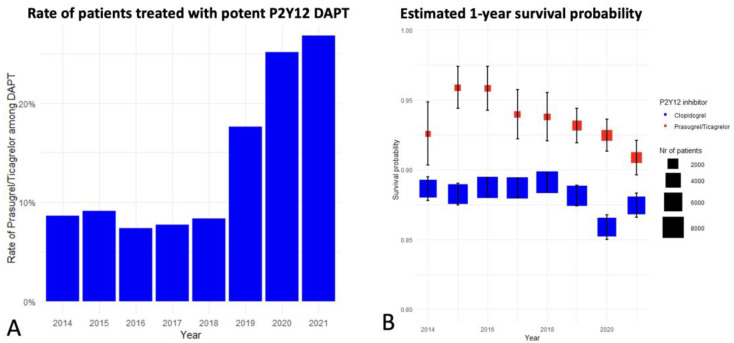
Annual Trends in Dual Antiplatelet Therapy (DAPT) Usage and Survival Outcomes among Patients with Myocardial Infarction Undergoing Coronary Intervention. The figure depicts the evolving landscape of DAPT in patients who experienced either ST-Elevation Myocardial Infarction (STEMI) or Non-ST-Elevation Myocardial Infarction (NSTEMI) and subsequently underwent Percutaneous Coronary Intervention (PCI), spanning the years 2014 to 2021. **Panel A**: illustrates the annual proportion of patients receiving potent P2Y12 inhibitors (prasugrel or ticagrelor) as part of their DAPT regimen. **Panel B**: presents the estimated 1-year survival probabilities stratified by the DAPT regimen using either clopidogrel or potent P2Y12 inhibitors (prasugrel/ticagrelor).

During the duration of the study, a significant upward trend was observed in the proportion of patients receiving potent P2Y12 inhibitors, culminating in these medications accounting for 25% of DAPT cases in the final year of 2021, as covered by our dataset (Figure 2A).

### 4.1. Mortality Analysis

**Figure 3:**
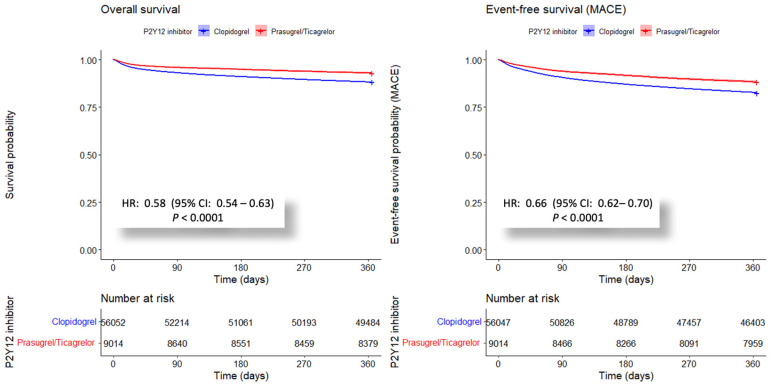
Comparative Kaplan–Meier Survival Analyses of Patients with STEMI or NSTEMI treated with PCI on Clopidogrel versus Potent P2Y12 Inhibitors. The figure illustrates the survival analysis for patients post-PCI caused by STEMI or NSTEMI, comparing the clopidogrel DAPT against potent P2Y12 inhibitors (Prasugrel/Ticagrelor)-based regimen. The left panel, “Overall survival”, depicts the survival probability over the first year following the intervention. The right panel, “MACE-free survival”, examines the span before the onset of major adverse cardiovascular events, including mortality, myocardial infarction, and stroke. Both segments incorporate risk tables, *p*-values for statistical comparison, and 95% confidence intervals, with clear color differentiation between the medication groups (Clopidogrel in blue; Prasugrel/Ticagrelor in red).

In the unadjusted analysis, the group receiving potent P2Y12 inhibitors exhibited a significantly lower risk of mortality within the first year after myocardial infarction, with an HR of 0.58 (95% CI: 0.54–0.63), compared to those on clopidogrel. (Figure 3). The absolute risk reduction in the first year was 4.75%, indicating that 21 cases needed to be treated to avoid one death. Annual trends showed that the benefits of potent DAPT remained significant alongside the increasing rate of its use. (Figure 2B) This finding was consistent with the IPTW analysis, which also showed a lower risk of mortality with an HR of 0.68 (95% CI: 0.65–0.71) for the potent P2Y12 inhibitor group (Table 2).

### 4.2. Major Adverse Cardiovascular Events (MACE) Analysis

Similar trends were observed in the incidence of MACE, which is defined as the composite of cardiovascular mortality, MI, and stroke. In the unadjusted model, the potent P2Y12 inhibitor cohort had a lower risk of MACE, with an HR of 0.66 (95% CI: 0.62–0.7) (Figure 3). The risk reduction at the end of the first year was calculated to be 5.57%, with an NNT of 18 to prevent one adverse event. This trend persisted in the IPTW analysis, with an HR of 0.73 (95% CI: 0.70–0.75) for the potent P2Y12 cohort (Table 2).

### 4.3. Additional Clinical Endpoints

Both the unadjusted and IPTW model analyses showed that the potent P2Y12 inhibitor group had a significantly lower risk of both myocardial infarction and stroke compared with the clopidogrel group. Interestingly, the analysis showed an increased rate of repeat revascularization in the potent P2Y12 group. This reached the level of statistical significance in both analyses because of the increased rate of PCI, whereas the differences in CABG were not significant. The increasing trend of repeat revascularization compared with the lower risk of ischemic events in the potent DAPT group might be explained by the higher rate of procedures with revascularization completion.

### 4.4. Subgroup Analysis

**Figure 4:**
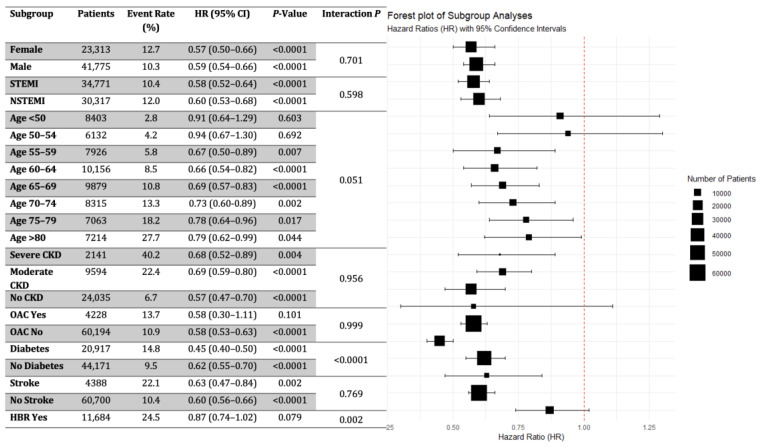
Subgroup Analysis of One-Year Mortality Risk: Comparison between Potent P2Y12 Inhibitors versus Clopidogrel. Cox regression analyses were performed in subgroups categorized by factors such as age, sex, presentation (STEMI or NSTEMI), diabetes status, stroke history, renal function (CKD status), and anticoagulant use (OAC). Based on the above parameters, patients were identified as having a high bleeding risk (HBR) status if they presented with at least one major or two minor ARC HBR criteria. The center of each square represents the hazard ratio (HR) for each subgroup. The size of the squares is proportional to the number of patients in each subgroup. The span of the horizontal lines represents the 95% Confidence Interval (CI) for HR. *p*-values indicate the statistical significance of HR for each subgroup. The *p*-interaction values assess the variability of treatment effects across subgroups.

Subgroup analyses revealed a generally favorable profile for potent P2Y12 inhibitors over clopidogrel in various patients undergoing PCI for AMI. Notable benefits were observed, particularly in patients with diabetes. The benefits of potent P2Y12 inhibitors varied across age groups. Significant reductions in risk were observed in individuals aged >55 years. However, in younger age groups (<50, 50–54), the treatment effect was not statistically significant, suggesting age-dependent variability in response to these therapies, particularly in these lower-risk categories. Importantly, in older age groups, the magnitude of this effect decreased, but significantly better outcomes were observed in potent DAPT-treated elderly patients. The treatment effect was consistent between STEMI and NSTEMI patients, with HRs of 0.58 and 0.60, respectively (Figure 4).

Diabetic patients benefited more markedly from potent P2Y12 inhibitors (HR: 0.45) than non-diabetic patients (HR: 0.62) with a significant interaction, highlighting the efficacy of these agents in this high-risk subgroup. The analysis revealed that risk was consistently reduced at all renal function levels. In the subgroup of patients with HBR status, a 13% reduction in mortality risk was observed; however, in this case, the effect of potent P2Y12 inhibitors did not reach statistical significance (HR: 0.87, *p* = 0.079). Subgroup analyses of MACE outcomes revealed similar results. (Supplementary Figure S1).

### 4.5. Decision-Curve Analysis (DCA)

**Figure 5:**
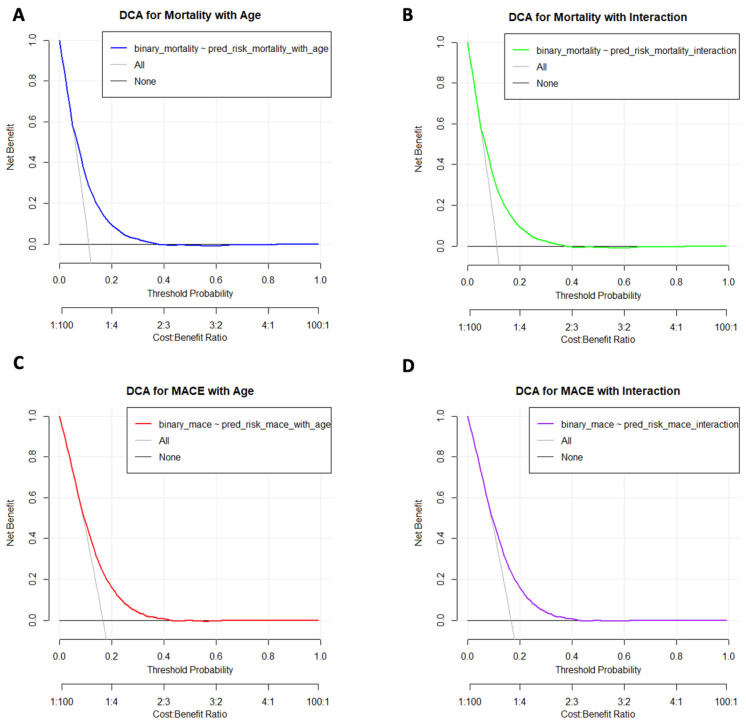
Decision-Curve Analysis of Mortality and MACE according to Age and Interaction Effects. Decision Curve Analysis (DCA) evaluating the net benefit of potent antiplatelet therapy for mortality and major adverse cardiovascular events (MACE). (**A**) DCA of mortality with age included as a continuous variable. (**B**) DCA of mortality with interaction between treatment and age. (**C**) DCA of MACE with age included as a continuous variable. (**D**) DCA of MACE with interaction between treatment and age. The net benefit is plotted against threshold probabilities, with models considering age (blue for mortality, red for MACE) and interaction terms (green for mortality, purple for MACE). The reference lines “All” and “None” represent the net benefits of treating all patients and treating none, respectively. The analysis showed that although age contributes to risk prediction, it does not significantly modify the treatment effect, supporting a uniform treatment approach across different age groups.

The inclusion of age as a continuous variable in the Cox models of mortality and MACE yielded a significant net benefit at low threshold probabilities. The net benefits for both mortality (Figure 5A) and MACE (Figure 5C) models declined rapidly and stabilized near zero around a threshold probability of 0.2. The interaction terms between treatment and age (Figure 5B,D) substantially modify the treatment effect of potent antiplatelets. These results indicate that although age should be considered in the overall risk assessment, treatment decisions based solely on age adjustments are not appropriate. Thus, a uniform approach to potent antiplatelet therapy across different age groups is needed to optimize clinical outcomes without requiring age-specific treatment modifications.

## 5. Discussion

Our study confirmed that in the context of DAPT following PCI for AMI, compared with clopidogrel, the use of potent P2Y12 inhibitors is associated with a reduced risk of mortality and MACE. In both the unadjusted and IPTW-adjusted models, patients treated with potent P2Y12 inhibitors exhibited a significantly lower risk of myocardial infarction and stroke compared to those receiving clopidogrel.

Upon analyzing the yearly stratified data from 2014 to 2021, a noteworthy observation was the consistently higher survival probability for patients treated with potent P2Y12 inhibitors compared to those receiving clopidogrel. This trend persisted annually, indicating a potentially significant association between potent P2Y12 inhibitor therapy and decreased absolute mortality within the first year after ACS. From 2019 to 2021, the COVID-19 pandemic introduced potential confounding factors affecting healthcare access and patient adherence, and the observed survival benefit persisted [16].

The subgroup analyses of our dataset provide nuanced insights pertinent to the 2023 ESC guidelines for ACS management. This is particularly relevant in the context of clopidogrel use among older patients with ACS and those with HBR. Our data indicate that for patients aged 75–79, the HR for mortality with potent P2Y12 inhibitors versus clopidogrel was 0.82 (95% CI: 0.69–0.96, *p* = 0.017), and for those aged >80 years, the HR was 0.79 (95% CI: 0.62–0.99, *p* = 0.044), suggesting significant reductions in mortality risk in both groups. It is noteworthy that the reduction in mortality remained significant even in the oldest age groups, despite the higher baseline mortality rate, which resulted in more avoidable events. Additionally, similar trends were observed for MACE. Combined with the DCA results showing that age contributes to risk prediction but does not significantly modify treatment effects, our findings support a uniform approach to antiplatelet therapy across all age groups. This challenges guideline recommendations that favor clopidogrel use in older patients, supporting the broad use of potent antiplatelets to optimize patient outcomes without age-specific adjustments [17,18].

For patients with HBR, the risk reduction implies that potent P2Y12 inhibitors may only provide a slight and statistically non-significant reduction in mortality compared with clopidogrel. This finding supports the guideline’s preference for clopidogrel in patients with HBR given the less clear benefit of potent inhibitors. Conversely, in patients with diabetes, we found that interaction *p* was highly significant for the diabetes status. This finding in the context of that the HR for mortality with potent P2Y12 inhibitors was 0.45 (95% CI: 0.40–0.50, *p* < 0.0001), indicating a substantial reduction in mortality risk compared with clopidogrel [19]. Although the smoker’s paradox and its potential interaction with the P2Y12 inhibitor response in STEMI patients warrant further study [20], this analysis lacked sufficient data to examine this relationship.

Furthermore, our results showed that patients with a history of stroke had more than twice the mortality rate compared with those without a cerebrovascular accident history. Notably, stroke is a contraindication to prasugrel, and recent stroke within the past six months is a major criterion in the ARC HBR definition [21]. In patients with a history of stroke, the HR was 0.63 (95% CI: 0.47–0.85, *p* = 0.0020), suggesting that clopidogrel was associated with increased mortality. However, our database lacks complete information to fully implement all ARC HBR criteria, including characteristics other than age, renal function, anemia, and stroke history.

Notably, an increased rate of repeat revascularization was observed in the potent P2Y12 inhibitor group. This increase was primarily attributable to the higher frequency of repeat PCI procedures, whereas the differences in CABG rates did not reach statistical significance. This finding, despite the lower ischemic event rates, may reflect a strategy to ensure complete initial revascularization.

P2Y12 and P2Y1 platelet receptors play critical roles in platelet activation and aggregation, influencing the development of thrombosis and cardiovascular events. The TRITON-TIMI 38 and PLATO trials assessed the efficacy of potent P2Y12 inhibitors in patients with acute coronary syndrome (ACS). Additionally, the ISAR REACT-5 trial provided a direct comparison between ticagrelor and prasugrel in patients with ACS undergoing invasive evaluation. Collectively, these studies have demonstrated that potent P2Y12 inhibitors are linked to a reduction in adverse thrombotic outcomes, including cardiovascular death, myocardial infarction, and stroke. However, it is crucial to note that although the PLATO trial reported a significant mortality benefit, this finding was not observed in patients with ACS treated with prasugrel. Significantly, the TRITON-TIMI 38 trials identified a ‘core population’ in which prasugrel demonstrated no benefit or potential harm. This population includes patients with a history of stroke or transient ischemic attack (TIA), as well as those aged >75 years or weighing less than 60 kg. Consequently, prasugrel’s labeling incorporates these findings, listing such cases as contraindications or recommending a lower dosage. This restriction may explain the more favorable outcomes observed with prasugrel in real-world applications than in trial settings. The on-label use of prasugrel, adhering to these specific guidelines, may also account for its superior performance in the ISAR-REACT-5 trial [22]. Notably, the incidence of stroke following coronary intervention was substantially reduced in patients administered potent DAPT, a trend that persisted and even intensified in analyses using IPTW. This finding supports the notion that stronger inhibition of platelet aggregation may effectively prevent stroke [23,24].

The cost and reimbursement policies of potent P2Y12 inhibitors significantly influence treatment decision making. The increasing popularity of de-escalation strategies, which involve switching to clopidogrel or P2Y12 monotherapy, also reflects concerns about the cumulative bleeding risk associated with prolonged potent DAPT [25,26,27,28,29,30,31]. Annual data analysis indicates a rising trend in the use of potent P2Y12 inhibitors, accompanied by sustained improvement in mortality. Despite the demonstrated benefits of potent P2Y12 inhibitors in reducing thrombotic risks among patients with ACS, their uptake in clinical practice has been gradual. Concerns regarding increased bleeding risks associated with the use of potent P2Y12 inhibitors are at the forefront of clinical decision making, particularly concerning medication choices at the time of hospital discharge. These risks necessitate a careful balance between reducing thrombotic events and avoiding bleeding complications [10].

## 6. Limitations

While offering valuable insights into the comparative effectiveness of potent P2Y12 inhibitors versus clopidogrel in patients with AMI undergoing PCI, this study has several limitations that necessitate cautious interpretation. The observational nature of the study introduces inherent selection bias, as the choice of antiplatelet therapy was at the discretion of the treating physician, influenced by factors (patient comorbidities, bleeding risk, resource availability) not fully captured in the dataset. Although statistical techniques like propensity score matching and inverse probability weighting were used to adjust for observed baseline differences between groups, residual confounding from unmeasured or inadequately adjusted factors remains a significant concern. Furthermore, the incomplete capture of all ARC high bleeding risk (HBR) criteria within the dataset limits the robustness and generalizability of findings related to this specific high-risk subgroup. The study’s reliance on a single national registry from Hungary restricts generalizability to other populations with differing healthcare systems, patient demographics, and treatment practices. The study period overlapped with the COVID-19 pandemic. Although efforts were made to account for its effects, potential confounding effects on healthcare access, resource allocation, treatment decisions, and patient outcomes cannot be fully excluded. In addition, the current study was limited by the lack of data regarding changes in P2Y12 therapy in-hospital or follow-up. Thus, we were not able to analyze potential treatment escalations (from clopidogrel to potent P2Y12 inhibitors) or de-escalations after the PCIduring hospitalization. This limitation may influence the interpretation of long-term outcomes. Finally, the extended seven-year follow-up period, while beneficial, might have introduced attrition bias; thus, we accounted for it by limiting the analysis of data for the first year after the ACS event. We performed the numerous post-hoc subgroup analyses that raise the risk of spurious findings due to multiple comparisons. Therefore, the results should be interpreted with caution, and further research, ideally a large-scale randomized controlled trial, is needed to validate these findings and draw more definitive conclusions regarding the optimal choice of antiplatelet therapy in this patient population.

## 7. Conclusions

In summary, our analysis indicated an association between the administration of potent P2Y12 inhibitors and improved survival in the year following ACS treatment, which was consistent across a seven-year period. This trend persisted even during the COVID-19 pandemic, although the precise implications of the pandemic on these outcomes warrant further analyses [32,33,34,35,36]. These findings underscore the potential importance of potent P2Y12 inhibitors in the management of patients with ACS. These findings reflect the importance of personalized medicine and the necessity of considering individual patient characteristics and comorbidities when selecting antiplatelet therapy for ACS management. Notable advantages in patients with diabetes and older age groups support the use of potent P2Y12 inhibitor-based DAPT. However, the decision to employ these agents in patients with HBR should be cautiously made considering individual patient profiles and associated risks. These findings may contribute to the evolving landscape of antiplatelet therapy in ACS management, emphasizing personalized treatment strategies to optimize patient outcomes.

## Figures and Tables

**Table 1:** Baseline Characteristics and Clinical History of Patients Undergoing Coronary Intervention with Dual Antiplatelet Therapy.

Variable	Clopidogrel (*n* = 55,958)	Potent P2Y12 Inhibitor (*n* = 9010)	*p* Value
Sex (Male)	35,755 (63.8%)	6020 (66.8%)	<0.01
Age (Year)	65.2 (±12.3)	61.6 (10.9)	<0.01
Heart Rate (1/min)	80.1 (±17.5)	80.8 (16.6)	<0.01
Blood Pressure (Systolic, mmHg)	135.6 (±24.3)	138.0 (24.6)	<0.01
Blood Pressure (Diastolic, mmHg)	80.0 (±14.2)	81.1 (14.2)	<0.01
Weight (kg)	81.2 (±17.1)	86.2 (17.6)	<0.01
Height (cm)	169.1 (±9.2)	170.4 (8.9)	<0.01
BMI (kg/m^2^)	28.4 (±7.4)	29.7 (5.7)	<0.01
Patient History			
Hypertension	43,241 (77.1%)	7085 (78.6%)	<0.01
Diabetes Mellitus (Type II)	16,165 (28.8%)	4752 (52.7%)	<0.01
Smoking	23,368 (41.7%)	3795 (42.1%)	0.44
KILLIP Class	I: 50,479 (90.4%) II: 3843 (6.9%) III: 989 (1.8%) IV: 533 (1.0%)	I: 8199 (91.1%) II: 575 (6.4%) III: 145 (1.6%) IV: 83 (0.9%)	0.22
ST-Segment Elevation Myocardial Infarction (STEMI)	29,508 (52.6%)	5263 (58.4%)	<0.01
Patient History			
Heart Failure	535 (9.6%)	554 (6.1%)	<0.01
Stroke	4068 (7.3%)	320 (3.6%)	<0.01
Peripheral Arterial Disease (PAD)	5881 (10.5%)	754 (8.4%)	<0.01
Elevated Serum Creatinine	10,560 (18.8%)	1451 (16.1%)	<0.01
Myocardial Infarction	8598 (15.3%)	1320 (14.6%)	0.11
Percutaneous Coronary Intervention (PCI)	8425 (15.0%)	1386 (15.4%)	0.37
Coronary Bypass Surgery (CABG)	2141 (3.8%)	269 (3.0%)	<0.01

**Table 2:** Cox Regression and IPTW Weighted Cox Regression Analyses of Clinical Endpoints in Patients Undergoing DAPT Post-Myocardial Infarction. Abbreviations: major adverse cardiovascular events (MACE), percutaneous coronary intervention (PCI), coronary artery bypass graft surgery (CABG), inverse probability treatment weighting (IPTW).

Clinical Endpoint	Overall Model	IPTW Adjusted Cox Regression Analysis
Mortality	0.58 (95% CI: 0.54–0.63)	0.68 (95% CI: 0.65–0.71)
MACE	0.66 (95% CI: 0.62–0.70)	0.73 (95% CI: 0.70–0.75)
Myocardial Infarction	0.82 (95% CI: 0.74–0.91)	0.7 (95% CI: 0.74–0.81)
Stroke	0.58 (95% CI: 0. 47–0.71)	0.76 (95% CI: 0.69–0.84)
Repeat Revascularization	1.2 (95% CI: 1.14–1.25)	1.09 (95% CI: 1.06–1.12)
Repeat PCI	1.21 (95% CI: 1.15–1.26)	1.1 (95% CI: 1.07–1.13)
CABG	1.1 (95% CI: 0.96–1.27)	0.97 (95% CI: 0.89–1.05)

## Data Availability

The data underlying this article will be shared upon reasonable request to the corresponding author.

## Supplementary Materials

The following supporting information can be downloaded at: https://www.mdpi.com/article/10.3390/jcm13216536/s1, Figure S1: Subgroup Analysis of One-Year MACE Risk: Comparison of Potent P2Y12 Inhibitors versus Clopidogrel; Figure S2: Inverse Treatment Probability Weighted Subgroup Analyses of One-Year Mortality and Major Adverse Event Risk: Comparison of Potent P2Y12 Inhibitors versus Clopidogrel.
