# Supplementary material for: The Comparative Effectiveness of Potent P2Y12 Inhibitors Versus Clopidogrel in Patients with Acute Myocardial Infarction Undergoing PCI: National Registry Data
Source: J Clin Med. 2024 Oct 30;13(21):6536. doi: 10.3390/jcm13216536 (PMC11546885; doi:10.3390/jcm13216536)

Supplementary material

to the article entitled **"The comparative Effectiveness of Potent P2Y12 Inhibitors Versus Clopidogrel in Acute Myocardial Infarction patients treated with primary PCI: National registry data"**

Contents

Supplementary material *1*

Figure S1 Subgroup Analysis of One-Year MACE Risk: Comparison of Potent P2Y12 Inhibitors versus Clopidogrel **2**

Figure S2 Inverse Treatment Probability Weighted Subgroup Analyses of One-Year Mortality and Major Adverse Event Risk: Comparison of Potent P2Y12 Inhibitors versus Clopidogrel **3**

Figure S1. Subgroup Analysis of One-Year MACE Risk: Comparison of Potent P2Y12 Inhibitors versus Clopidogrel

Cox regression analyses were performed in subgroups categorized by factors such as age, sex, presentation (STEMI or NSTEMI), diabetes status, stroke history, renal function (CKD status), anticoagulant Use (OAC). Patients based on the above parameters were identified as having High Bleeding Risk (HBR) status if presenting with at least one major or two minor ARC HBR criteria. The center of each square represents the hazard ratio (HR) for each subgroup. The size of squares is proportional to the number of patients in each subgroup. The span of the horizontal lines represents the 95% Confidence Interval (CI) for HR. P-values indicate the statistical significance of the HR for each subgroup. The P-interaction values assess the variability of treatment effects across subgroups.

Subgroup	Patients	Event Rate (%)	HR (95% CI)	P-Value	Interaction P
Female	23313	18.6	0.64 (0.57-0.71)	<0.001	0.404
Male	41775	15.5	0.67 (0.62-0.73)	<0.001	
STEMI	34771	15.0	0.65 (0.60-0.71)	<0.001	0.44
NSTEMI	30317	18.5	0.68 (0.62-0.75)	<0.001	
Age < 50	8403	6.6	0.79 (0.63-1.01)	0.06	0.337
Age 50-54	6132	8.4	0.87 (0.69-1.11)	0.26	
Age 55-59	7926	10.4	0.81 (0.67-0.99)	0.042	
Age 60-64	10156	14.0	0.74 (0.64-0.87)	<0.001	
Age 65-69	9879	16.7	0.78 (0.68-0.90)	<0.001	
Age 70-74	8315	19.7	0.74 (0.63-0.87)	<0.001	
Age 75-79	7063	25.0	0.82 (0.69-0.96)	0.017	
Age >80	7214	33.8	0.77 (0.62-0.96)	0.018	
Severe CKD	2141	46.6	0.75 (0.59-0.95)	0.018	0.973
Moderate CKD	9594	28.9	0.75 (0.66-0.86)	<0.001	
No CKD	24035	12.1	0.70 (0.61-0.80)	<0.001	
OAC Yes	4228	20.0	0.61 (0.36-1.04)	0.068	0.808
OAC No	60194	16.3	0.66 (0.62-0.70)	<0.001	
Diabetes	20917	21.9	0.51 (0.47-0.56)	<0.001	<0.001
No Diabetes	44171	14.1	0.68 (0.61-0.74)	<0.001	
History of Stroke	4388	31.5	0.74 (0.59-0.92)	0.008	0.49
No Stroke	60700	15.5	0.68 (0.63-0.72)	<0.001	
HBR Yes	11684	31.1	0.93 (0.81-1.06)	0.269	<0.001

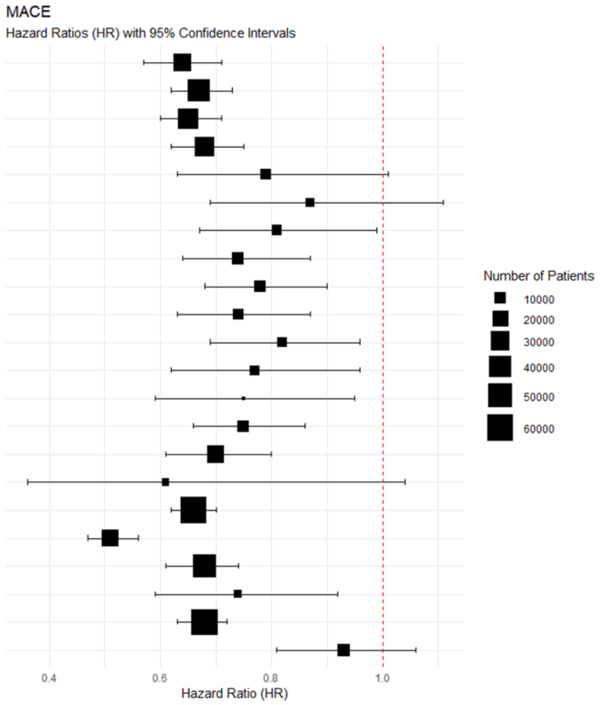

Figure S2. Inverse Treatment Probability Weighted Subgroup Analyses of One-Year Mortality and Major Adverse Event Risk: Comparison of Potent P2Y12 Inhibitors versus Clopidogrel

Cox regression analyses were performed in subgroups categorized by factors such as age, sex, presentation (STEMI or NSTEMI), diabetes status, stroke history, renal function (CKD status), anticoagulant Use (OAC). Patients based on the above parameters were identified as having High Bleeding Risk (HBR) status if presenting with at least one major or two minor ARC HBR criteria. The center of each square represents the hazard ratio (HR) for each subgroup. The size of squares is proportional to the number of patients in each subgroup. The span of the horizontal lines represents the 95% Confidence Interval (CI) for HR. P-values indicate the statistical significance of the HR for each subgroup. The P-interaction values assess the variability of treatment effects across subgroups.

	Mortality	Major Adverse events
Subgroup	HR (95% CI)	HR (95% CI)
Female	0.65 (0.54 - 0.79)***	0.65 (0.56 - 0.75)***
Male	0.72 (0.63 - 0.83)***	0.78 (0.70 - 0.87)***
STEMI	0.69 (0.59 - 0.80)***	0.73 (0.65 - 0.83)***
NSTEMI	0.70 (0.59 - 0.83)***	0.72 (0.63 - 0.82)***
Age < 50	0.98 (0.63 - 1.53)	0.69 (0.50 - 0.94)*
Age 50-54	1.02 (0.69 - 1.51)	1.03 (0.77 - 1.37)
Age 55-59	0.78 (0.53 - 1.13)	0.82 (0.63 - 1.06)
Age 60-64	0.63 (0.48 - 0.84)***	0.68 (0.55 - 0.83)***
Age 65-69	0.76 (0.60 - 0.98)*	0.84 (0.69 - 1.01)
Age 70-74	0.69 (0.53 - 0.90)**	0.68 (0.54 - 0.85)***
Age 75-79	0.72 (0.53 - 0.98)*	0.79 (0.62 - 1.00)
Age >80	0.74 (0.53 - 1.03)	0.68 (0.50 - 0.93)*
Severe CKD	0.70 (0.48 - 1.04)	0.81 (0.58 - 1.12)
Moderate CKD	0.67 (0.53 - 0.84)***	0.72 (0.59 - 0.88)***
No CKD	0.64 (0.50 - 0.83)***	0.74 (0.62 - 0.89)***
OAC Yes	0.63 (0.28 - 1.44)	0.58 (0.29 - 1.14)
OAC No	0.70 (0.62 - 0.78)***	0.73 (0.67 - 0.80)***
Diabetes	0.57 (0.49 - 0.66)***	0.63 (0.56 - 0.70)***
No Diabetes	0.79 (0.67 - 0.93)**	0.80 (0.71 - 0.91)***
History of Stroke	0.72 (0.47 - 1.09)	0.76 (0.55 - 1.06)
No Stroke	0.68 (0.61 - 0.77)***	0.72 (0.65 - 0.78)***
HBR Yes	0.83 (0.64 - 1.07)	0.88 (0.71 - 1.09)

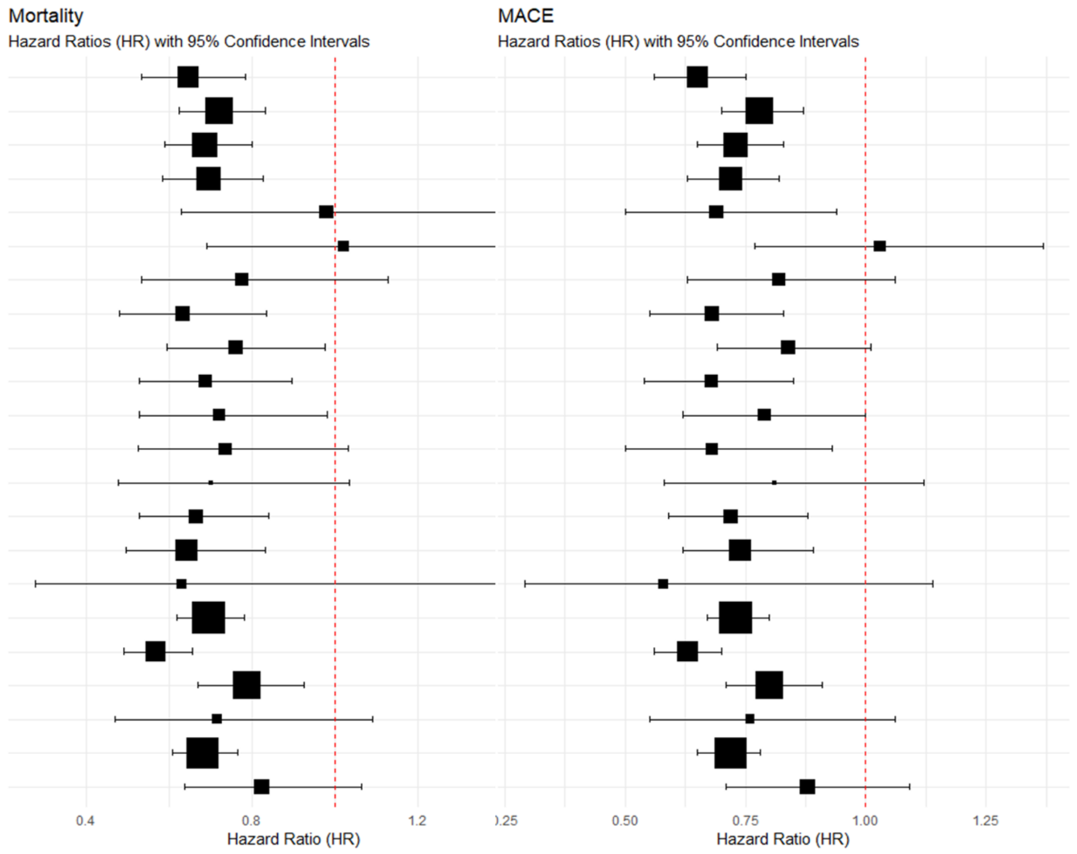

Supplement: Supplementary file 1 [file jcm-13-06536-s001.zip › jcm-3239663-supplementary.pdf]
